# Bilateral Leydig Cell Tumor in a Capuchin Monkey (*Sapajus apella*): Case Report

**DOI:** 10.1111/jmp.70118

**Published:** 2026-09-28

**Authors:** Ewerton Lourenço Barbosa Favacho, Sandla Freitas da Silva, Jamerson Luiz Brito da Silva, Daniella Kaísa de Oliveira Bezerra, Valíria Duarte Cerqueira, Gabriela Riet Correa Rivero, Pedro Soares Bezerra Júnior

**Affiliations:** ^1^ Animal Pathology Laboratory (LAPATO) Federal University of Pará (UFPA), Graduate Program in Animal Health in the Amazon (PPGSAAM) Castanhal Pará Brazil; ^2^ Ministry of Health, Secretariat of Health Surveillance and Environment National Primate Center Ananindeua Pará Brazil; ^3^ Experimental Primate School (EEP) Federal University of Pará (UFPA) Belém Pará Brazil

**Keywords:** nonhuman primates, reproductive pathology, testicular neoplasia

## Abstract

Leydig cell tumors (LCTs) are uncommon in nonhuman primates (NHPs). A bilateral LCT was identified in a 20‐year‐old male capuchin monkey ( *Sapajus apella* ) maintained under human care. Ultrasonography revealed a right testicular nodule, while necropsy identified bilateral testicular masses. Histopathology confirmed bilateral LCT. This case documents bilateral LCT in *S. apella* , a rare finding in NHPs.

## 1 Introduction

Testicular neoplasms in NHPs are uncommon and have been reported in only a few individuals and as isolated cases [1, 2, 3, 4, 5, 6, 7]. Reports of bilateral LCTs in NHPs are scarce [4, 6], with few cases documented in primates of the genus Sapajus [5, 7]. The present study describes the clinical and pathological features of an LCT in a capuchin monkey ( *Sapajus apella* ) maintained under human care, with the aim of contributing to knowledge of this type of tumor in NHPs.

## 2 Materials and Methods

### 2.1 Humane Care Guidelines

The animal was maintained under human care at the Escola Experimental de Primatas (EEP). The present study used exclusively material obtained after the animal's death and did not involve experimental procedures, interventions, or experimentation on live animals.

### 2.2 Case Description

A male capuchin monkey ( *Sapajus apella* ), approximately 20 years old, maintained under human care at the Escola Experimental de Primatas (EEP), had a history of enlargement of the right testicle since 2023. The animal underwent ultrasonographic examination, which revealed an enlarged right testicle (3.18 cm), with heterogeneous echotexture, mixed echogenicity, and an anechoic cystic structure measuring 0.98 × 0.57 cm, suggesting a degenerative or infiltrative process of possible neoplastic origin. The left testicle had preserved dimensions (2.3 cm) (Figure 2B). Cytological examination was performed, and the findings were consistent with a hemorrhagic cystic formation, with no evidence of neoplastic cells.

In 2024, the animal died as a result of neurological abnormalities and was submitted for necropsy at the Animal Pathology Laboratory of the Federal University of Pará (UFPA), Castanhal Campus. On external examination, both testes were located in their normal scrotal position and were enlarged. The right testicle contained a friable nodule measuring 3.5 × 2.0 cm, orange in color on cut surface, with cystic cavities filled with clear fluid. The left testicle showed a similar but smaller nodule, measuring 2.1 × 1.0 cm (Figure 1). No significant gross lesions were observed in other organs.

**FIGURE 1:**
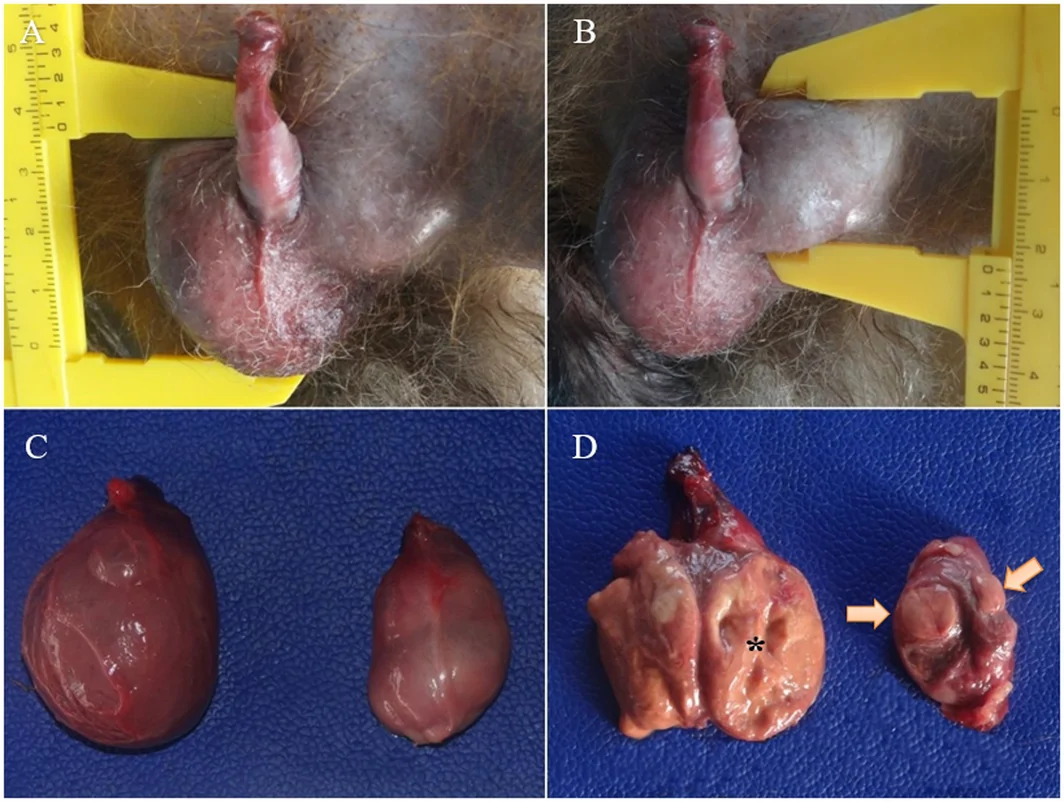
Gross findings in a capuchin monkey ( *Sapajus apella* ). (A–C) Enlargement of the right and left testes, measuring 3.5 × 2.0 cm and 2.1 × 1.0 cm, respectively. (D) Cut surface with an orange coloration, showing a well‐demarcated, friable mass associated with cavities filled with clear fluid (*) and well‐demarcated nodules of variable sizes (arrow).

Fragments of various organs, including the testes, were collected, fixed in 10% buffered formalin, routinely processed for histology, embedded in paraffin, sectioned at 5 μm thickness, and stained with hematoxylin and eosin (H&E), according to the protocol described by Prophet et al. [8].

Histologically, the testicular masses were composed of pleomorphic cells arranged in a solid pattern and supported by scant stroma. The cells had abundant eosinophilic cytoplasm containing numerous vacuoles of variable sizes and occasional brownish pigment. The nuclei were round and homogeneous, with dense chromatin and inconspicuous nucleoli, and no mitotic figures were observed in 2.37 mm^2^. Cystic areas were also observed, containing hyaline, mildly eosinophilic material, with no cells identified within the cystic spaces. No associated hemorrhage or large accumulations of blood were observed. In the remaining testicular parenchyma, marked depletion of germ cells within the seminiferous tubules and mild interstitial fibrosis were observed. The epididymal tubules were empty (Figure 2C,D). Based on the microscopic findings, a histopathological diagnosis of bilateral Leydig cell tumor was established.

**FIGURE 2:**
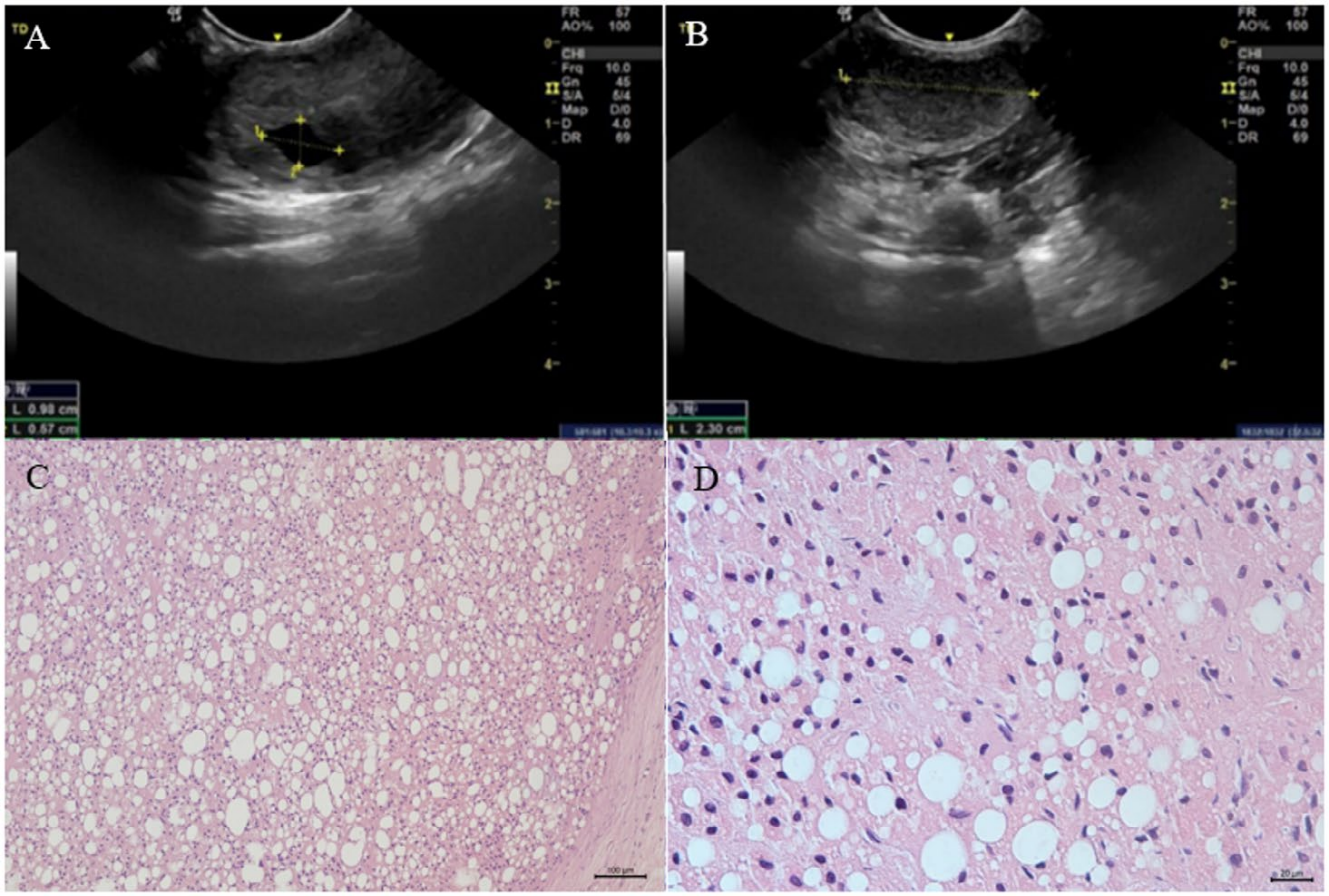
Ultrasonographic and histopathological findings in a capuchin monkey ( *Sapajus apella* ). (A) Enlarged right testicle (3.18 cm), showing heterogeneous echotexture, mixed echogenicity, and a cystic structure measuring 0.98 × 0.57 cm. (B) Left testicle with preserved dimensions (2.3 cm) and homogeneous echotexture. (C) Proliferation of pleomorphic neoplastic cells arranged in a solid pattern, supported by scant stroma, with abundant eosinophilic cytoplasm containing multiple vacuoles of variable sizes (10×). (D) Neoplastic proliferation at higher magnification (40×).

## 3 Discussion

The diagnosis of bilateral Leydig cell tumor in the present study was established based on the pathological findings, which were consistent with those previously described in the literature for NHPs [4, 5, 6, 7]. However, in this case, LCT was confirmed in both testes, differing from previous reports in NHPs, in which this tumor was identified in only one testicle. In those reports, although tumors were present in both testes, they originated from different cell types, with LCTs restricted to the left testicle [5, 6, 7]. In humans, these tumors show no preference for laterality, with bilateral occurrence reported in approximately 3% of cases [9, 10].

In NHPs, LCTs are poorly documented, and both their incidence and the factors involved in their development remain poorly established [7, 11]. Although cryptorchidism represents a predisposing factor in humans, this type of tumor is not associated with this condition in other species, and several possible causes related to its development have been described [12, 13, 14, 15, 16]. In this context, despite the phylogenetic proximity to humans, no association between cryptorchidism and tumor development was observed, and the etiopathogenic mechanisms involved in these species remain uncertain.

Endocrine manifestations associated with LCTs are poorly described in NHPs. Although alopecia has been reported as a sign of hyperestrogenism in other species [17], in the present case it was not possible to determine whether this finding resulted from the neoplasm or senescence. Signs of feminization were not observed, differing from those described in humans [13], possibly influenced by the animal's cohabitation with a neutered female, which made libido assessment difficult. Therefore, it was not possible to differentiate tumor‐associated hyperestrogenemia from effects related to senescence, and the presence of subclinical hormonal activity cannot be ruled out.

Scrotal ultrasonography was a rapid, noninvasive, and low‐cost method and was useful for evaluating the right testicular nodule. However, it had limitations in assessing the contralateral testicle, reinforcing the importance of correlating ultrasonographic findings with gross and histopathological findings. In human medicine, ultrasonography is widely used because of its availability, painless nature, and favorable cost‐effectiveness, and it is employed in the investigation of testicular disorders, including nodules, masses, and enlargement [18, 19]. Furthermore, intratesticular masses should be considered potentially malignant, making histopathological confirmation necessary [20].

Although cytological examinations may serve as a diagnostic tool, their sensitivity for detecting LCTs is limited because these tumors generally grow slowly and rarely cause enlargement. In addition to cystic areas that may compromise sample adequacy, as probably occurred in the present study, most of these tumors are identified incidentally during necropsy [15, 16, 17, 21, 22].

It is important to highlight that the main differential diagnoses for LCT in NHPs include other primary testicular neoplasms, such as seminoma and Sertoli cell tumor,1–3,5,7 as well as non‐neoplastic conditions such as testicular hyperplasia [6]. However, the histological features observed in the present case are considered sufficient for definitive diagnosis in both humans [23] and other animal species [24].

LCTs in NHPs generally exhibit benign behavior and have a favorable prognosis, with orchiectomy being the treatment of choice [5, 24]. However, surgical intervention was not indicated in the present case because clinical management was directed toward treating the neurological condition presented by the animal. In addition, no evidence of metastasis was observed during necropsy, supporting the generally favorable biological behavior described for this neoplasm.

## Funding

This study was partially supported by the Coordination for the Improvement of Higher Education Personnel—Brazil (CAPES), Finance Code 001, during the author's Master's degree (2026–present). The author was also awarded a scholarship by the Brazilian Ministry of Education (MEC) to undertake the Animal Pathology Residency Program at the Federal University of Pará from 2024 to 2026.

## Ethics Statement

The authors confirm that the ethical policies of the Journal of Medical Primatology were followed. Ethical approval was not required because the study used exclusively samples obtained from animal cadavers, with no procedures or experimentation involving live animals and was exempted from review by the Animal Ethics Committee of the Federal University of Pará (CEUA‐UFPA), protocol No. 20260625. The use of the samples was authorized by the Chico Mendes Institute for Biodiversity Conservation (ICMBio) through the Biodiversity Authorization and Information System (SISBIO), protocol No. 103613, in accordance with the regulations of the Brazilian National Council for the Control of Animal Experimentation (CONCEA).

## Conflicts of Interest

The authors declare no conflicts of interest.

## Data Availability

The data supporting this study are available from the corresponding author upon request.

## References

[1] A. Gozalo , T. Nolan , and E. Montoya , “Spontaneous Seminoma in an Owl Monkey in Captivity,” Journal of Medical Primatology 21 (1992): 39–41. 1602459

[2] C. A. Maruffo , “Spontaneous Tumours in Howler Monkeys,” Nature 213, no. 5075 (1967): 521. 10.1038/213521a0 4962854

[3] H. M. Hayes and B. Sass , “Testis Neoplasia in Captive Wildlife Mammals: Comparative Aspects and Review,” Journal of Zoo Animal Medicine 18, no. 4 (1987): 162–165.

[4] W. B. Karesh , M. S. Burton , R. G. Russell , and M. W. Burns , “Leydig Cell Tumor in a Western Lowland Gorilla ( *Gorilla gorilla gorilla* ),” Journal of Zoo Animal Medicine 19 (1988): 51–54.

[5] G. Fraess , R. Dickinson , B. Kragness , and M. Sadar , “Synchronous Bilateral Testicular Neoplasms in a Black‐Capped Capuchin ( *Sapajus apella* ),” Veterinary Record Case Reports 7 (2019): e000841.

[6] S. Kirejczyk , C. Pinelli , S. Gumber , O. Gonzalez , S. Kumar , and E. J. Dick, Jr. , “Urogenital Lesions in Nonhuman Primates at 2 National Primate Research Centers,” Veterinary Pathology 58 (2021): 147–160. 10.1177/0300985820971752 PMC7903448 33208023

[7] F. A. D. De Holleben Camozzato , E. T. Schulz , E. A. Costa , et al., “Seminoma and Interstitial Leydig Cell Tumor in a Capuchin Monkey ( *Sapajus nigritus* ),” Comparative Clinical Pathology 34 (2025): 509–514.

[8] E. B. Prophet , B. Mills , J. B. Arrington , and L. H. Sobin , Laboratory Methods in Histotechnology (Armed Forces Institute of Pathology, 1992).

[9] I. Kim , R. H. Young , and R. E. Scully , “Leydig Cell Tumors of the Testis: A Clinicopathological Analysis of 40 Cases and Review of the Literature,” American Journal of Surgical Pathology 9 (1985): 177–192. 10.1097/00000478-198503000-00002 3993830

[10] M. M. Muheilan , M. Shomaf , E. Tarawneh , M. M. Murshidi , M. R. Al‐Sayyed , and M. M. Murshidi , “Leydig Cell Tumor in Grey Zone: A Case Report,” International Journal of Surgery Case Reports 35 (2017): 12–16. 10.1016/j.ijscr.2017.03.043 PMC5394201 28419904

[11] A. Moresco , Y. Feltrer‐Rambaud , D. Wolfman , and D. W. Agnew , “Reproductive One Health in Primates,” American Journal of Primatology 84 (2022): e23325. 10.1002/ajp.23325 34516669

[12] H. Lejeune , R. Habert , and J. M. Saez , “Origin, Proliferation and Differentiation of Leydig Cells,” Journal of Molecular Endocrinology 20 (1998): 1–26. 10.1677/jme.0.0200001 9513078

[13] O. M. Al‐Agha and C. A. Axiotis , “An In‐Depth Look at Leydig Cell Tumor of the Testis,” Archives of Pathology & Laboratory Medicine 131 (2007): 311–317. 10.5858/2007-131-311-AILALC 17284120

[14] C. Pozza , R. Pofi , M. Tenuta , et al., “Clinical Presentation, Management and Follow‐Up of 83 Patients With Leydig Cell Tumors of the Testis: A Prospective Case‐Cohort Study,” Human Reproduction 34 (2019): 1389–1403. 10.1093/humrep/dez083 PMC6688875 31532522

[15] F. O. Conrado and S. E. Henderson , “Testes, Ovaries, and Prostate,” in Veterinary Cytology (Wiley, 2020), 499–530.

[16] L. Solano‐Gallego and M. Masserdotti , “Reproductive System,” in Canine and Feline Cytopathology: A Color Atlas and Interpretation Guide, 4th ed., ed. R. E. Raskin , D. J. Meyer , and K. M. Boes (Saunders, 2022), 440–484.

[17] C. R. Daleck and A. B. De Nardi , Oncologia em cães e gatos, 2nd ed. (Editora Roca, 2016), 766.

[18] A. L. Kühn , E. Scortegagna , K. M. Nowitzki , and J. H. Kim , “Ultrasonography of the Scrotum in Adults,” Ultrasonography 35 (2016): 180–197. 10.14366/usg.15075 PMC4939719 26983766

[19] F. Maxwell , A. Savignac , O. Bekdache , et al., “Leydig Cell Tumors of the Testis: An Update of the Imaging Characteristics of a Not So Rare Lesion,” Cancers 14 (2022): 3652. 10.3390/cancers14153652 PMC9367522 35954321

[20] S. J. Gaddam , F. Bicer , and G. T. Chesnut , “Testicular Cancer,” in StatPearls (StatPearls Publishing, 2023). 33085306

[21] D. W. Agnew and N. J. MacLachlan , “Tumors of the Genital Systems,” in Tumors in Domestic Animals, 5th ed., ed. D. J. Meuten (John Wiley & Sons, 2017), 689–722.

[22] A. G. Burton , “Reproductive,” in Clinical Atlas of Small Animal Cytology and Hematology, 2nd ed., ed. A. G. Burton (Wiley‐Blackwell, 2024), 345–375.

[23] M. Crabtree , D. Cheng , and L. Barajas , “Testicular Leydig Cell Tumor in a 64‐Year‐Old Man With Cytological High‐Grade Features and no Metastasis,” Human Pathology: Case Reports 23 (2021): 200464.

[24] D. J. Meuten , ed., Tumors in Domestic Animals (John Wiley & Sons, 2020).

